# Primitive Reflex Factors Influence Walking Gait in Young Children: An Observational Study

**DOI:** 10.3390/ijerph19074070

**Published:** 2022-03-29

**Authors:** Ewa Gieysztor, Mateusz Kowal, Małgorzata Paprocka-Borowicz

**Affiliations:** Department of Physiotherapy, Faculty of Health Sciences, Wroclaw Medical University, 50-367 Wrocław, Poland; mateusz.kowal@umw.edu.pl (M.K.); malgorzata.paprocka-borowicz@umw.edu.pl (M.P.-B.)

**Keywords:** primitive reflexes, children, spatio-temporal parameters, gait

## Abstract

Background: Primitive reflexes (PRs) are observed as an automatic response to a specific stimulus. They are vivid from intrauterine life to 6 months postnatal. The reactions are inhibited with the growing maturation of the central nervous system (CNS). In some cases, when the natural process of development is incorrect, PRs manifest later. The analysis of differentiation in gait parameters in children with persistent PRs is important for better understanding their specific behaviour and movement. This study’s aim was to investigate the influence of active PRs on the gait parameters of preschool children. Methods: There were 50 children examined, 30 girls and 20 boys. They were 3.5–6 years old. The children had persistent PRs in the trace form. Each child was examined by S. Goddard’s Battery Test. The acquisition of the spatial-temporal gait parameters was performed using a BTS G-SENSOR measurement instrument. Participants walked barefoot, in the most natural way for them, at a self-selected speed on a 5 m walkway, then turned around and went back. They performed this twice. Results: The reflex activity influences gait cycle duration (*p* = 0.0099), the left step length (*p* = 0.0002), the left double support phase (*p* = 0.0024), the right double support phase (*p* = 0.0258) and the right single phase. Difficulties in recreating the crawling pattern and GRASP reflex influence gait cadence (*p* < 0.05). The left GRASP reflex corresponds to step length (*p* < 0.05). The activeness of the symmetrical tonic neck reflex correlates with the right single support (*p* < 0.05). Conclusion: The presence of PRs affect walking gait in preschool children.

## 1. Introduction

The phenomenon of an immature central nervous system (CNS) can be observed in some preschool children. This is especially noticeable in the activeness of primitive reflexes (PRs). PRs are an automatic response to a specific stimulus. They manifest in utero and should have desisted by the sixth month of life. No activity of the reflexes is expected. The reflexes are restrained due to the growing maturation of the central nervous system. In some cases, when the natural process of development is incorrect, PRs can manifest later in life. PR activeness is increasingly observed, even in groups of neurologically healthy children [1,2,3,4]. In subjects with CNS injuries, such as cerebral palsy (CP), the full range of PR reactions is visible. The trace form of PRs is observed in children, later in life, usually of preschool or school-age and diagnosed, for example, as ADHD or specific learning difficulties [1]. Primitive reflexes indicate the level of neuromotor immaturity in children [5,6,7]. Some studies show the association of motor developmental delays with the activeness of primitive reflexes (APR), along with posture development [4,8]. Children with APR move in a specific way during spontaneous play and walking (gait). They have no neurological and musculoskeletal impairments, but their movement patterns may be affected by muscle tension caused by the reflex’s response [9]. The gait may be influenced by many factors. The most obvious are injuries of the lower limbs or brain injuries, but the subtle changes in the gait smoothness may also be noticed in children with gentle disturbances in brain activity, which may be observed in the activeness of primitive reflexes. There are no previous studies of gait parameters in children with APR; however, gait analyses in children with ADHD or CP are common and they are similar for comparison purposes because it is proven that some PRs are also active in children with ADHD [10,11,12].

Gait factors such as duration (s), cadence (steps/min), velocity (m/s), step length (m) and step length to height component, as well as gait cycle duration (s), step length (%), support phase duration (%), swing phase (%), double support duration (%) and single support duration (%) are usually analysed [13,14,15,16]. The surveying of the differentiation in gait parameters in children with APR is important for better understanding their specific behaviour and movement in preschool diagnoses and specialist medical diagnoses, but also in the natural environment of the child. The patterns of the gait and spontaneous movement of the child with APR are often described using qualitative and subjective methods (e.g., descriptive method) [17,18,19]. Unfortunately, such a process does not allow for detailed knowledge of the spatio-temporal variables in the gait cycle, so more precise analyses cannot be performed without specific human movement analysis and the application of devices.

Taking the above into consideration, the basic aim of the study was to investigate the impact of primitive reflexes on walking gait.

## 2. Materials and Methods

### 2.1. Study Design

The observational study is part of the PRACS (Primitive Reflexes and All Children Sphere) project. The project investigates primitive reflexes and their impact on the motor, sensory, and cognitive development in preschool and school-age children. There are four articles published from the project so far [20,21,22].

The research related to human use has complied with all relevant national regulations and institutional policies, following the tenets of the Declaration of Helsinki. The study was approved by the Wroclaw Medical University’s Ethical Committee KB-116/2019. All parents of the subjects were kept informed of the purpose and process of examination and had given their written consent prior to the study.

### 2.2. Definitions

Crawling—crawling pattern

PR—primitive reflexes

ATNR R and L—asymmetrical tonic neck reflex, right and left sides

ATNR in standing—ATNR examined by Schilder test

STNR FLX and EXT symmetrical tonic neck reflex in flexion and in extension

GALANT R and L—Galant reflex

TLR FLX and EXT—tonic labyrinthine reflex in flexion and in extension

MORO—Moro reflex

GRASP P and L—Grasp reflex, right and left side

### 2.3. Participants

**Scheme 1:**
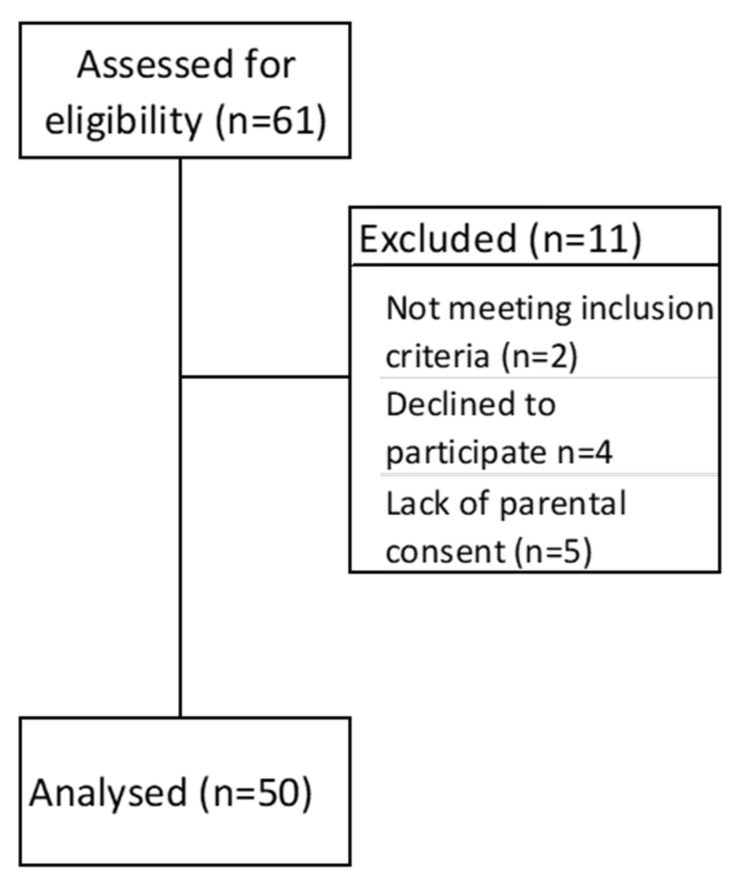
Flow chart of participation.

Preschool children whose parents/guardians consented to the study were included in the study. The inclusion criteria were communicativeness at the level of command understanding and proper motor and intellectual development of the child. The examination took place in the preschool of the child in a known environment. The flow chart of participation is shown in Scheme 1.

**Figure 1:**
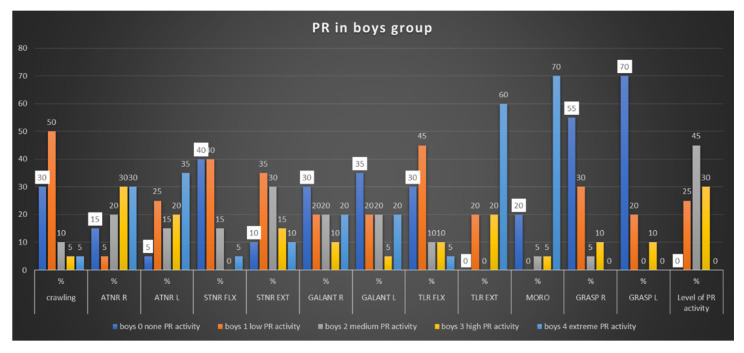
The scale of primitive reflexes (PRs) activity in boys. Percentage of crawling pattern performance and the reflexes activity.

**Figure 2:**
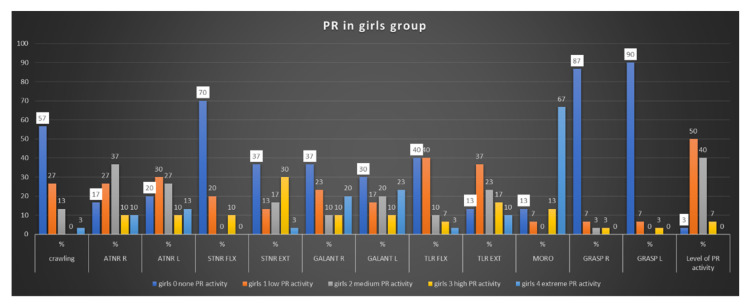
The scale of primitive reflexes (PRs) activity in girls. Percentage of crawling pattern performance and the reflexes activity.

Fifty children aged 3.5–6 years old were examined, 30 girls and 20 boys. The mean age of the group was 5.5 (±0.5) years. The mean height of the children was 100 cm (±10) and the mean weight was 17 kg (±3.4). The children had APR in trace form covering the entire spectrum of intensity–from none to alarming, according to the S. Goddard scale [6]. The specific characteristics of the degree of PR activity, divided by gender, are presented in Figure 1 and Figure 2.

**Figure 3:**
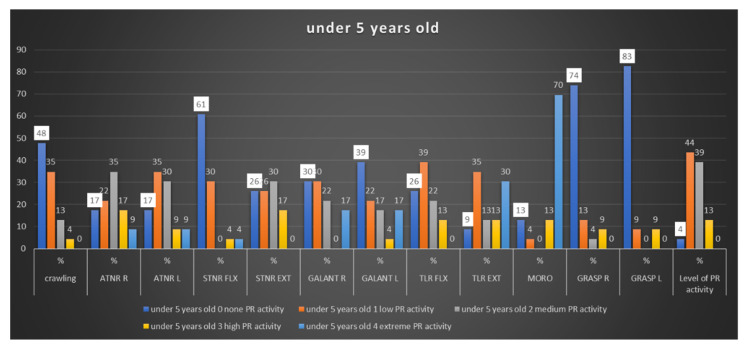
The scale of primitive reflexes (PRs) activity in younger children. Percentage of crawling pattern performance and the reflexes activity.

**Figure 4:**
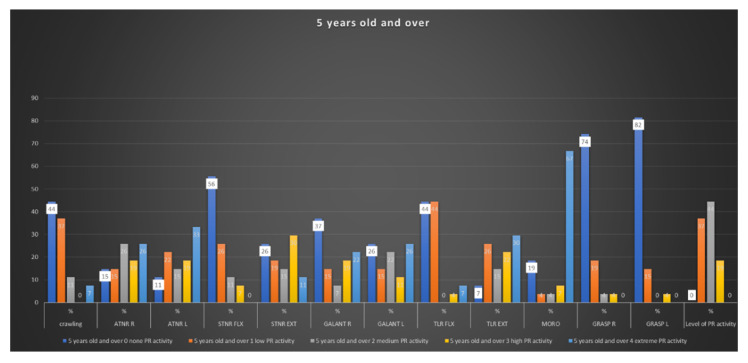
The scale of primitive reflexes (PRs) activity in older children. Percentage of crawling pattern performance and the reflexes activity.

Moreover, the characteristics of the children due to age are presented in Figure 3 and Figure 4.

The percentage of PR occurrence in groups of girls and boys is presented in Figure 1 and Figure 2. Of the boys, 75% achieved a higher score of PR activity. In the group of girls, this was under 50%. The least integrated were the Galant Reflex (GR) and Moro Reflex (MR) in the group of younger children, and Tonic Labyrinthine Reflex (TLR) and MR in the group of older children.

There were two significant differences in the group of examined children. Boys had higher scores in TLR in extension (EXT) than girls (*p* = 0.028), which means a higher level of TLR activity, while younger children had Romberg with eyes closed at a higher activity level (*p* = 0.038).

### 2.4. Measurement

INPP Battery Test by S. Goddard [6].

Each child was examined by S. Goddard’s Battery Test. The tests covered: Crawling, Romberg test, Asymmetrical Tonic Neck Reflex (ATNR), Symmetrical Tonic Neck Reflex (STNR), Tonic Labyrinthine Reflex (TLR), Galant Reflex (GR), Moro Reflex (MORO), Grasp reflex (GRASP).

For establishing the primitive reflexes profile, the points of ATNR left and right, STNR in flexion and extension, TLR in flexion and extension, GR left and right and MR were summed up. The results were then divided into the activity of the reflexes in five levels:

0—no reflex activity; 

1—low reflex activity; 

2—medium reflex activity; 

3—high reflex activity; 

4—alarming reflex activity.

The testing protocol used followed Gieysztor et al. [4].

### 2.5. Spatio-Temporal Gait Parameters

For the acquisition of the spatial-temporal gait parameters, a BTS G-SENSOR (BTS Bioengineering Corp., Quincy, MA, USA) was used. The instrument was equipped with Triaxial Accelerometer 16 bit/axes with multiple sensitivity (±2, ±4, ±8, ±16 g), Triaxial Gyroscope 16 bit/axes with multiple sensitivity (±250, ±500, ±1000, ±2000 °/s), as well as Triaxial Magnetometer, 13 bit: (±1200 μT). The G-sensor is suitable for assessing physical activity, as shown by analyses of the inter-instrument correlation coefficient from 0.90 to 0.99 and the coefficient of variation between instruments ≤2.5% [16,23].

Before the measurement, each child performed the same exercises (warm-up) in order to prepare better for the test. The measurement was performed in an environment known to children. Body height and leg length (from the greater trochanter to the floor) were measured prior to gait measurement which was performed by placing a wireless inertia sensor on the lower lumbar region. The gauge was centred on the L4–L5 intervertebral disc. Participants walked barefoot, in the most natural way for them, at a speed of their choice along a five-meter pavement, turning and back twice. The raw data was then processed using the Smart Analyser G-Walk (BTS Bioengineering, Quincy, MA, USA), devoted software to calculate spatio-temporal parameters such as: cadence (steps/min), velocity (m/s), step length (m), swing and double support phase duration (calculated as a percentage of the gait cycle).

### 2.6. Sample Size Calculation

The study sample size (*n* = 50) was sufficient to achieve 80% power assuming the correlation coefficient threshold being equal to 0.4, and the two-tailed alpha level-0.05 (calculation performed with the use of the G * Power 3.1.9.7.).

### 2.7. Statistical Analysis

Statistical analysis was performed using IBM SPSS Statistics version 25. For continuous variables, arithmetic means and standard deviations were calculated. Moreover, Spearman’s rho correlation analysis was used in order to determine the relationship between quantitative variables. To compare two groups in terms of quantitative/ordinal variables Mann–Whitney’s U test was used. The analysis of the results for the right and left side gait parameters was made with the Wilcoxon labelled rank test. The chi-square test of independence was used to compare groups in terms of nominal/categorical variables. The level α = 0.05 was used for all comparisons. The power of the test was estimated using the G * Power 3.1.9.4 program. for the analysis of correlation, with the assumed sample N = 50 and α = 0.05. The test power was 0.57. For the comparison of age groups, the power of the test was 0.73.

## 3. Results

The gait results, such as means, standard deviations and medians of spatio-temporal parameters of gait in the group were collected in Table 1.

### 3.1. Gait Parameters and Retained Primitive Reflexes Correlations

Some of the reflexes influence most gait parameters. The coefficient of determination R^2^ was calculated for the model with one variable, in turn for each of the variables. The percentage distribution is shown in Table 2. This summary shows that STNR FLX and ATNR R in standing affect almost all of the spatio-temporal gait parameters.

In order to check the relationship between retained reflexes and gait parameters, an analysis of Spearman’s rho was carried out.

The analysis showed a positive relationship between the duration of the gait and Tonic Labyrinthine Reflex FLX. Cadence is negatively correlated with the level of reflex activity, crawling and the GRASP reflex of the right hand. Children with higher levels of reflex activity took longer steps during gait analysis. A longer step was observed in children who had difficulty recreating the crawling pattern, while the right GRASP reflex increased.

The presence of the right GRASP reflex positively correlates with the duration of the gait analysis. There is also a positive correlation between the left GRASP reflex and step length, and a negative correlation between the left GRASP reflex and the right step length. In children with left GRASP reflex, the left step is extended, while the right step is shortened.

The duration of the single right support phase correlates positively with the STNR in extension. Children with a higher Symmetrical Tonic Neck Reflex EXT indicator have a longer phase of single support on the right leg.

Table 3 and Table 4 show the results.

### 3.2. Gait Parameters Divided into Two Groups Due to Age and Gender

An analysis of the correlation between age and gait parameters in the examined children was performed. It showed significant relationships between the age and duration of the double support phase (*p* = 0.005), the duration of the single support phase (right) (*p* = 0.025), and the developed steps for the left and right legs (*p* < 0.03). In older children, the duration of the double support phase is shorter and the number of steps smaller, while the duration of the single support phase (right) is longer.

Gait parameters were also compared according to age. Children under 5 years of age obtained a higher statistically significant result for cadence (*p* = 0.017), duration of the double support phase (*p* = 0.005) and number of steps (*p* = 0.002). Older children were statistically significant for a longer duration of the single support phase (right side) (*p* = 0.011) and gait duration for the right side (*p* = 0.031). The differences in gait parameters between boys and girls were also calculated. The Mann–Whitney U test shows no statistically significant differences between genders in the examined group.

## 4. Discussion

Searching new aspects of child development, working in new fields and using new tools to understand children in the process of growing up is one of the goals of the PRACS project. We analysed the neuromotor development of the children in the aspects of physical, social and educational status [8,20,21,22,24]. The presented article shows the results of the research connected with spatio-temporal gait parameters. The children with APR are often perceived as those who cannot remain in one place for a long time. Their gestures are chaotic, and often uncoordinated. Functionally they are still in the norm, but the manner of execution is a bit different than typical. It seems this is because children with neuromotor delay have central muscle stability impairment, which causes higher peripheral muscle tension [21].

In this research, we evaluated the gait parameters in a group of typically developed children within neuro-maturity disturbance cases, manifested in the persistence of primitive reflexes. The gait analysis shows the next aspect of child functioning.

Symmetrical Tonic Neck Reflex FLX and Asymmetrical Tonic Neck Reflex R are very common reflexes observed in the examined group. In the study, we found that the activity of these two reflexes influenced almost all of the studied gait parameters (78% of all analysed). This means that the persistence even of a single reflex can modulate the parameters of walking gait in children. STNR is frequently observed in children with ADHD symptoms [9]. It is known that these children have a specific way of moving, i.e., ‘they usually are everywhere’. Their specific walk is sometimes described as uncoordinated or clumsy. This effect may be explained by the results of our study. Moreover, seeing the power of the cumulative effect of the reflexes on child functioning, we could predict larger gait abnormalities when reflexes overlap each other, even if they are of very low intensity. As such, our study covered many more PRs.

As mentioned above, the gait analysis of ADHD children can function as a measure of comparison to our findings. This is because in the group of children with ADHD we have found some variability of gait connected with the degree of PR activity, especially in terms of step length or single support phase time. In children with neuro-maturity disorders, the gait variability was observed by Manicolo et al. [25]. Their study exhibits irregular gait patterns and greater variability in the timing of steps in children with ADHD. In addition, the authors emphasize that the results may indicate a delay in puberty rather than a permanent deviation from gait. They show that gait patterns tend to become more regular with age. Referring to this statement, we would expect better regularity in gaits of children with APR with age caused by growing maturation, as shown by Gieysztor et al. [26]. The variability of gait is commonly observed in children with APR, as well as ADHD, and are both popularly referred to as clumsiness. Some authors analysed the gait symmetries in children with ADHD and developmental coordination disorders. Many gait variables in these groups were observed by the authors, but some of the spatio-temporal gait variables did not differ from the control group [27,28,29]. These abnormalities are approximated in this study, where we tried to indicate and name some differences in the movement patterns of children with APR. More objective research is, however, necessary to describe variability in clumsy behaviour. At the same time, we expect that our research will constitute a springboard for the wider investigation of the neuro-maturity disorders manifested, for example, in APR.

In our study, cadence was negatively correlated with the level of PR activity. Children with a higher level of PR activity had lower cadence. Longer walking steps in those children may be seen as clumsy, but this form of gait coexisted with difficulty in recreating the crawling pattern. Papadopoulos et al. [28], who studied the gait of ADHD children during self-selected fast speed, show that they had higher cadence and walked faster than the control group. The examination shows some change in gait pattern, which corresponds to a timing deficit. Dziuba et al. [15] conducted gait analysis in children with cerebral palsy (CP), who usually exhibit PR at a higher level than children with neuromotor delays [30]. The results showed that the speed of the gait in some groups of children with CP does not differ from healthy children, with some moving even faster. Then, there were the results of children with mild CP and good overall functional status. This study underlines the difficulty in comparing results, caused by the different forms of CNS damage. Another research study conducted by Deconinck et al. [14] compared 10 subjects of Developmental Coordination Disorder (DCD) to 10 typically developed (TD) children. They showed shorter strides in both time and space during stepping. This gait pattern was characterised by a higher frequency than their TD peers. In our study, the distance of the gait took more time for children with higher TLR FLX. For this particular reflex activity, the result was different than in the above-mentioned study.

Furthermore, in our study, we found that asymmetry in gait patterns is linked with the occurrence of reflexes on one side. The results show that in children with the left GRASP reflex, the left step is extended, and the right step is shortened. The muscle arousal in the region of the upper limb can transfer tension in the region of the lower extremities. Another difference in the results was the longer single support phase on the right lower limb in children with a higher STNR EXT indicator.

ADHD-participants, in the study of Buderath et al. [23,31], presented gait and balance abnormalities and deficient coordination of postural adjustments during tasks.

In our study, the analysis of spatio-temporal gait parameters due to the age of the examined group shows the correlation resulting from the growth of children. In older children, the duration of the double support phase was shorter, the number of steps smaller, and the longer step and duration of the single support phase (right) longer. Comparing children under and over 5 years of age shows significant differences in some spatio-temporal gait parameters. The cadence and double support phase time, as well as the number of steps, had higher values in the younger group. Moreover, gait duration time and single support phase on the right foot had lower values in this group. Similar results describing an older group of participants (5 to 13 years old) were obtained by Lythgo et al. [29]. They assessed the gait in three different speeds and indicated the significant speed differentiation between slow, free and fast conditions. The examined pupils walked 24% slower and 30% faster than the free speed condition. The Lythgo et al. results encouraged us to conduct the gait study in the group of children with APR in various conditions such as: speed, environment, footwear. In this context, Moreno-Hernandez et al. [32] also provided some suggestions. They argue that the variation in gait patterns may depend on the footwear. The use of footwear resulted in an increase in speed, cadence, stride and stride length, while the percentage of the support phase decreased. The authors also found no significant differences between gender. The same results were found in our study.

All of the above-mentioned measurements were performed using various motion analysis software. The available literature does not allow for a strict comparison of the results due to the different methods of data collection; however, it paints a concrete picture of the subject. The lack of studies analysing detailed primitive reflexes is the reason why it is difficult to compare the results. All studies used for discussion can only point to similarities in the gait, but in spite of different points of view, the comparison cannot be strict. Moreover, it is obvious that there are many more items that should be taken into consideration in gait analysis [33].

Our study presents the significant influence of reflexes on walking gait in preschool children. The findings can explain some abnormalities in the group of children with neurodevelopmental delays. They are the first reports on the subject, which refer to primitive reflexes. As PR can be observed in paediatrics, the findings can be important for paediatricians and physical therapists working with developmentally delayed children, as well as teachers and parents, and highlight a reason for the abnormal movements of children. Knowing the cause of the walking abnormality, there is scope to expand the treatment on primitive reflexes therapy with complex results. The study shows that PR activity is worth examining, as it is much more meaningful than previously acknowledged.

Limitations of the study:

Gait analysis was based on time–space measurements of gait variables only. Such analysis does not provide a complete picture of movement in children with active PR. Future research is essential to assess changes in the pelvis, torso and skull during gait movement, and ground responses due to reflex evaluation concerning Asymmetrical Tonic Neck Reflex, Symmetrical Tonic Neck Reflex, Tonic Labyrinthine Reflex, Galant Reflex and Moro Reflex. Such tests, however, provide clinical guidelines and extend the biomechanical knowledge necessary to assess a child’s development and work on supporting its development. The advantage of such an examination may be clinically viable and tangible.

## 5. Conclusions

The presence of primitive reflexes affects the spontaneous motion of children, as expressed in their gait. Symmetrical and Asymmetrical Tonic Neck Reflex are the most frequent reflexes whose activeness correlates with gait parameters. Taking into account the impact of primary reflexes on a child’s motor skills suggests difficulties in neuromotor maturation and may clarify the ‘bit weird’ way of movement in children with neuromotor disturbances. For practitioners, this may be valuable information for the diagnosis and treatment of children with neurodevelopmental immaturity.

## Figures and Tables

**Table 1:** Spatio-temporal parameters of gait in children with APR.

Parameter	Mean	SD	Median	95% CI
analysis duration (s)	44.8	10.6	43.7	41.7–4.7
cadence (steps/min)	136.6	20.7	132.8	130.7–142.6
velocity (m/s)	0.8	0.3	0.8	0.7–0.9
step length (m)	0.7	0.2	0.8	0.6.–0.9
% step length/height	65.8	21.3	69.3	60–72
gait cycle duration (s)				
left	1.0	0.3	0.9	0.9–1.1
right	1.0	0.4	0.9	0.9–1.1
step length (%)				
left	51.7	7.9	50.5	49.4–54.0
right	48.3	7.9	49.5	46.0–50.6
support phase duration (%)				
left	66.7	4.1	66.4	65.5–67.9
right	65.6	4.4	65.4	64.3–66.9
swing phase (%)				
left	33.3	4.1	33.6	32.1
right	34.4	4.4	34.6	34.5
double support duration (%)				
left	19.4	21.7	15.7	33.1
right	15.4	3.3	15.8	35.6
single support duration (%)				
left	34.4	3.9	34.0	33.3–35.5
right	33.9	4.1	33.7	32.7–35.1
steps analysed				
left	9.1	5.1	8.5	7.6–10.5
right	9.5	5.5	9.0	8.0–11.1

CI—Confidence Interval; m—metre; m/s—metre per second; steps/min—steps per minute.

**Table 2:** Distribution of frequency in the influence of gait parameters by some of the examined reflex factors.

Reflex Parameter	Value of Influence	Gait Parameter
STNR FLX	78%	
ATNR R in standing	78%	
MORO	67%	
PR activity	67%	all gait parameters
ROMBERG open	56%	
crawling	44%	
GALANT L	39%	

STNR FLX–Symmetrical Tonic Neck Reflex in flexion; ATNR R in standing—asymmetrical tonic neck reflex (right side) examined by Schilder test; MORO—Moro reflex; PR activity—Primitive Reflex activity as the sum of the reflexes; ROMBERG open—Romberg test with eyes open; crawling—performance of crawling pattern; GALANT L—Galant reflex left side.

**Table 3:** Spearman’s correlations between selected reflexes and gait parameters such as duration, cadence, velocity, step length and proportion of step length and height.

Kind of Reflex/Test	Analysis Duration	Cadence	Velocity	Step Length	% Step Length/Height
crawling	0.140	−0.318 *	−0.120	0.020	0.062
TLR FLX	0.297 *	−0.236	0.059	0.181	0.210
GRASP R	0.195	−0.300 *	−0.147	−0.033	0.045
Level of PR activity	0.052	−0.291 *	−0.009	0.158	0.122

* *p* < 0.05; crawling—performance of crawling pattern; TLR FLX—Symmetrical Tonic Neck Reflex in flexion; GRASP R—Grasp reflex right side; Level of PR activity—Primitive Reflex activity as the sum of the reflexes.

**Table 4:** Spearman’s correlations between selected reflexes and gait parameters are mostly divided into left and right side. Gait time, step length, stance and swing phase time, double and single support phase time, along with some of the primitive reflexes’ correlations are shown.

Gait Parameter/Kind of Reflex	STNR EXT	GALANT R	GRASP R	GRASP L
Gait time				
left	0.130	−0.018	0.286 *	0.087
right	0.137	0.014	0.238	0.071
Step length				
left	0.203	−0.218	0.245	0.325 *
right	−0.138	0.218	−0.245	−0.325 *
Stance phase time				
left	−0.138	−0.094	−0.167	−0.166
right	−0.093	−0.024	−0.187	−0.094
Swing phase time				
left	0.138	0.094	0.167	0.166
right	0.093	0.024	0.187	0.094
Double support phase time	−0.262	0.172	−0.148	0.006
Single support phase time				
left	0.162	−0.136	0.156	0.065
right	0.289 *	−0.097	0.083	<0.001

STNR EXT—Symmetrical Tonic Neck Reflex in extension; GALANT R—Galant reflex right side; GRASP R and L—Grasp reflex right and left sides; * *p* < 0.05.

## Data Availability

The data presented in this study are available on request from the corresponding author.
